# Guided web-based treatment program for reducing cannabis use: a randomized controlled trial

**DOI:** 10.1186/s13722-020-00185-8

**Published:** 2020-02-18

**Authors:** Kristina Sinadinovic, Magnus Johansson, Ann-Sofie Johansson, Thomas Lundqvist, Philip Lindner, Ulric Hermansson

**Affiliations:** 1 Centre for Psychiatry Research, Department of Clinical Neuroscience, Karolinska Institutet & Stockholm Health Care Services, Region Stockholm, Stockholm, Sweden; 2 Department of Global Public Health, Karolinska Institutet, Stockholm, Sweden; 3 Stockholm Center for Dependency Disorders, Stockholm Health Care Services, Region Stockholm, Stockholm, Sweden; 4 Department of Psychology, Lund University, Lund, Sweden; 5 Department of Psychology, Stockholm University, Stockholm, Sweden

**Keywords:** Cannabis, Web-based treatment with therapist guidance, Cognitive behavioral treatment, eHealth, Randomized controlled trial

## Abstract

**Background:**

The aim of this study was to investigate the effects of a web-based treatment program with therapist guidance for adults and adolescents with regular cannabis use from the general population.

**Methods:**

A double blinded randomized controlled trial with a parallel group design was conducted (intervention group n = 151, wait-list control group n = 152). Follow-up 12 weeks from treatment commencement of a 13-module intervention. The primary outcome was frequency of cannabis use. Time by group interaction effects were modeled using generalized estimated equations and the instrumental variable approach was used to estimate the effect of intervention adherence.

**Results:**

At follow-up, the intention to treat (ITT) analyses did not show any significant time by group effects. A significant association between intervention adherence and scores on the cannabis abuse screening test (CAST) was found. Secondary analysis excluding participants who had received other professional help revealed time by group effects for secondary outcomes gram cannabis consumed past week, number of dependency criteria and CAST score. Due to methodological limitations, these latter results should be interpreted with caution.

**Conclusions:**

In this study we did not find a web-based treatment program with therapist guidance to be more effective than a waiting-list in reducing frequency of cannabis use.

*Trial registration* The trial was pre-registered at ClinicalTrials.gov (NCT02408640) April 3, 2015

## Background

Globally, cannabis is the most commonly used psychoactive substance under international control [1–3]. The demand for treatment of cannabis-use disorders and associated health conditions in high- and middle-income countries is increasing [1]. Treatment guidelines for cannabis abuse or dependence emphasize immediate abstinence, during which psychosocial treatment should be initiated. Although effective treatment alternatives are available for individuals wanting to quit their cannabis use [1, 4], research shows only a small proportion of individuals who meet criteria for cannabis abuse or dependence seek professional help [5].

Increasing interest in internet-based interventions designed to help individuals reduce or end cannabis use likely indicates efforts to meet the wide range of needs related to treatment of cannabis use. According to WHO, internet-based interventions for cannabis-use disorders is an area of priority for future research [1]. Internet-based interventions, in the context of substance use, represents supportive interventions with the aim of helping users reduce or end their consumption of the particular substance [6]. Compared to a regular informational website, an internet-based intervention is more structured and provides a variety of interactive support, e.g., for self-monitoring. Mechanisms for support can be preprogrammed text and/or direct communication, with, e.g., a therapist, via e-mail, chat or bulletin boards [7].

### Previous studies

Few published studies have examined effects of internet-based interventions based on cognitive and behavioral approaches for reducing or ending cannabis use and targeting the general population. Present results are promising but also show methodological limitations with low adherence to treatment and low follow-up rates.

A solution-focused internet-based treatment program, consisting of 50 days of diary-writing coupled with weekly contact with a therapist via a synchronous chat, was shown to reduce frequency and quantity of cannabis use as well as levels of anxiety and depression and more so compared to no treatment. In the treatment group, 360 of 860 (40%) received the intervention and 206 of the 1292 randomized participants (16%) were included in the analysis [8]. A further study did not show any differences in effects of a shortened program or when the chat-based counseling option was removed [9].

A fully automated internet-based self-help program with six modules drawing on cognitive-behavioral therapy (CBT) and motivational interviewing (MI), with no therapist support reduced both frequency and quantity of cannabis use more than a program of psycho-educational materials organized in six internet-based modules at 6-week and 3-month follow-ups. A mean of 3.5 of the 6 modules were completed in the intervention group and 122 of 225 (51%) completed the 3-month follow-up [10].

Schaub and colleagues (2015) showed that an eight-module internet-based self-help program, based on MI and CBT with the opportunity for chatting with a therapist was more effective in reducing the number of days with cannabis use, compared to the same program without opportunity for chat and compered to no intervention at 3 months follow-up. In addition, a higher proportion of participants in the self-help group with opportunity for chat, compared to the group with no opportunity for chat, reported no use of cannabis at follow-up. No differences were detected between self-help group without opportunity for chat and the group with no intervention in days of cannabis use or participants with no use at follow-up. The chat group completed a mean of 3.2 out of 8 modules and 23.7% received at least one chat session. At 3 months 117 out of 308 (38.0%) participants could be followed up [11].

Copeland and colleagues (2017) showed an equal reduction of frequency and quantity of cannabis use as well as severity of cannabis dependence at 1 month follow-up, irrespective of whether study participants received a brief or an extended version of individualized feedback, both generated in the context of a brief web-based intervention of motivational enhancement [12]. In a small, low-powered (n = 38) and non-randomized comparative study, Budney and colleagues (2011) could not find differences in attendance and retention in treatment, nor cannabis use, irrespective of whether study participants received an internet-based 12-week intervention of nine treatment sessions of motivational enhancement therapy (MET), CBT and contingency management (CM) or face to face delivery of the same intervention content [13].

In summary, the few previously published studies and several meta-analyses [14–16] show that internet-based interventions might reduce cannabis use. However, more research is needed to build an evidence base for the effects of such interventions. In the current study shorter modules and extra reinforcement for completing follow-up were used in an effort to improve follow-up rates and treatment adherence.

### Aim and hypotheses

The aim of this study was twofold. Given that internet-based interventions represent a new way to target cannabis users in Sweden, one purpose was to investigate whether it is possible to reach regular cannabis users via a web-based treatment program. The second purpose was to investigate the effects of the Swedish web-based treatment program with therapist guidance, Cannabishjälpen, designed to help adolescents and adults from the general population to reduce or end cannabis use. We hypothesized that, in comparison to no intervention, Cannabishjälpen would be associated with greater reduction in cannabis consumption, cannabis-related consequences, and greater degree of help-seeking.

## Method

In order to test our hypotheses, an RCT was conducted using a parallel group design with a 1:1 allocation ratio. Recruitment and randomization of study participants as well as data collection at both baseline and follow-up were conducted anonymously and entirely online. Similarly, the treatment program tested as part of this trial was delivered exclusively via the internet. This study was approved by the Stockholm Regional Ethical Review Board (No. 2014/1374-31/5) and was pre-registered at ClinicalTrials.gov (NCT02408640).

### Setting and recruitment procedure

**Fig. 1:**
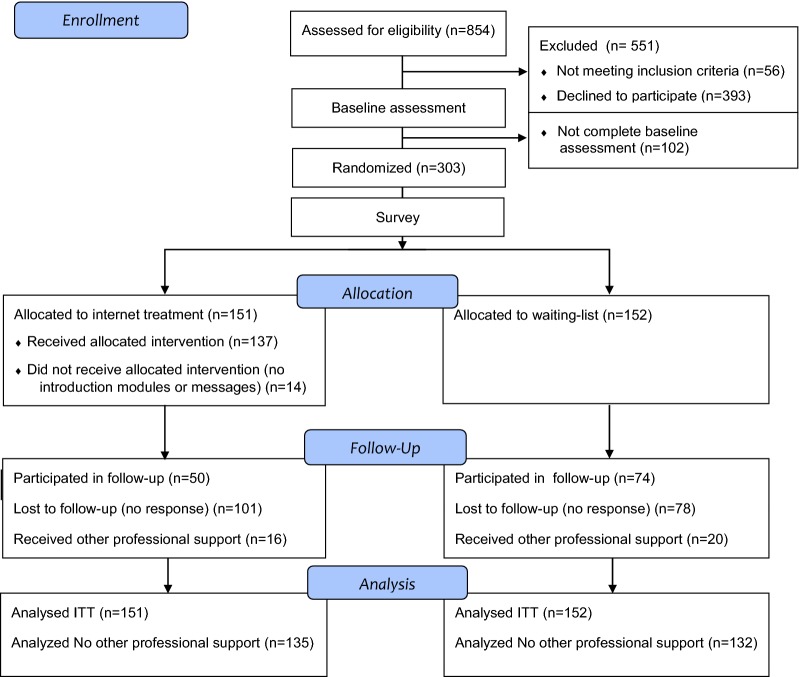
CONSORT 2010 flow diagram

Individuals recruited were visitors to a Swedish informational website (http://www.cannabishjalpen.se) seeking information or help for issues related to cannabis use. The website was advertised through social media, search engines, cannabis-related websites, paper ads and flyers. The website had a mean of 13,314 (SD = 2389) unique monthly visitors during the recruitment period and approximately 25% of visitors entered pages that were aimed at cannabis users. Individuals interested in internet interventions for changing their cannabis use were invited to participate in this study. Thus, study participants were self-selected and active help-seekers prior to intervention onset. Recruitment was conducted between June 2015 and June 2017, individuals who were interested in participating in the study provided their informed consent after being informed about the study. In order to blind study participants, they were informed that the aim was to investigate which internet-based services should preferably be made available for persons wishing to reduce or end their cannabis use, and, to examine whether such services are helpful to change their cannabis use. A total of 854 screening forms, indicating interest in participating in the study were filled out, 580 by men (68%) and 274 by women (32%). A flowchart illustrating the study participation process is presented in Fig. 1.

In this initial assessment, potential study participants answered questions constituting the first part of the baseline measurements. They responded to questions regarding gender, age, country of birth, education, employment, marital status and housing conditions. Regarding cannabis use, they answered one question about the frequency of their cannabis use during the past 6 months and completed a calendar where they registered which days, of the past week, they had used cannabis and how much cannabis they had used during the week in grams. In addition, they identified negative consequences of their cannabis use, by completing the assessment instruments Cannabis Abuse Screening Test (CAST) [17, 18] and DSM-5 criteria for cannabis use disorder [19]. Finally, they responded to one question regarding their motivation to change their cannabis use [20].

### Eligibility criteria

Individuals, 16 years or older, who had used cannabis at least once a week during the last 6 months were eligible to participate in the study. Before being given access to the treatment program, study participants were informed that the program was not intended for use by individuals with a current or past psychosis, schizophrenia, bipolar disorder and/or ongoing suicidal thoughts. Furthermore, participants were informed that individuals unable to read and write in Swedish would not be able to benefit from the program.

### Baseline measurement

In order to continue, individuals who met eligibility criteria were requested to create a user account on the internet-based study platform, by registering a username, a password and an email address. Subsequently, they completed a seven-day Time Line Follow Back (TLFB) where they registered which days in the past week they had used alcohol and how many standard drinks they had consumed on each occasion [21, 22] and questions about their use of illicit drugs other than alcohol and cannabis. Study participants further responded to questions targeting depression by completing Montgomery Åsberg Depression Rating Scale—Self reported (MADRS-S) [23, 24], anxiety by completing the Generalized Anxiety Disorder Assessment (GAD-7) [25] and Sense of Coherence scale (SCS) [26, 27]. Finally, they responded to whether they had received any professional help to reduce or end their use of cannabis in the past 12 months.

### Randomization

Study participants were randomized, in blocks of 20, by a fully automated allocation system programed in the websites content management system Drupal. Researchers were blinded regarding group assignment. Participants were blinded to what intervention the other group received and they were informed that they would receive access to try an intervention at some time point after they had completed the survey.

### Study arms

After randomization, all study participants filled out a survey regarding, in their opinion, which internet-based services for individuals who wish to reduce or end their cannabis use should be available and how such services should preferably be designed. Upon completion of these questions, participants allocated to the intervention group were given access to the web-based treatment program.

#### Intervention

The web-based treatment program consisted of psychoeducative information based on a manual-based treatment program for chronic cannabis users, *A way out of fog* [28], as well as training and exercises grounded on principles of Cognitive Behavioral Therapy and Motivational Interviewing.

Initially, a therapist, working with the program, sent a welcome message to each user including short personal feedback about his or her baseline assessments. Subsequently, participants had the opportunity to communicate with the therapist throughout the entire program, on their own terms.

The program included 13 modules (see Table 3 for details) and a calendar, in which the participants registered which days they had used or not used cannabis. The program was divided into several short modules in an effort to promote completion. Each module consisted of short text sessions and questions. Users could also choose to listen to the module being read by one of the therapists. All modules were available to the participants at once, with the recommendation of doing one or two modules per week during the 6 weeks period. However, participants received automated recommendations about which modules to work with, based on the duration of time since they last used cannabis, along with personal feedback regarding their information in the calendar. In addition to the automated recommendation the therapist could recommend modules based on communication with the user.

#### Wait-list control group

Individuals allocated to the control group went through exactly the same procedure as the individuals allocated to the intervention group, with the difference that they were given access to the treatment program only after completing the 3-month follow-up.

Participants in both groups had access to facts, frequently asked questions and information about how to access treatment for cannabis use disorders, via the start page of Cannabishjälpen.

### Follow-up procedure

Three months after the recruitment to the study, participants from both groups were invited via email to participate in the 3-month follow-up, consisting of questions about cannabis and alcohol use, depression and anxiety, seeking professional help to reduce or end cannabis use, and, asking relatives or friends for help for the same purpose. In addition, participants were asked whether they had used any internet- or telephone-based services in order to reduce or end their cannabis use.

Individuals who did not respond to the follow-up received a total of six reminder emails with 5-day intervals. As an attempt to improve the follow-up rate, all study participants who completed the 3-month follow-up were included in a raffle, where 1 out of 25 received an iPad, which they were informed about after randomization.

### Outcome measures

The primary outcome measure for this study was the number of days without cannabis use in the past 7 days. Secondary outcome measures included: estimated grams of cannabis consumed in the past 7 days, self-rated number of DSM-5 criteria for cannabis use disorder during the past 3 months, CAST-score, proportion of study participants seeking professional help to reduce or end cannabis use since entering the study, number of standard (alcohol) drinks consumed in the past 7 days, SCS, MADRS-S and GAD-7, respectively.

### Sample size

An a priori power calculation revealed that n = 176 participants per group would be required to detect a d = 0.3 (small-medium) between-group, post-treatment effect size with 80% power.

### Statistical analyses

Generalized estimating equations (GEE), with robust standard errors and exchangeable correlation structures, were used to model time × group effects (full factorial models) on each outcome measure separately. These models included all available data (consistent with the Intention To Treat, ITT, principle) and used the appropriate family function (Poisson, Gaussian or binomial). In studies with few repeated measures, mixed effects modeling is inappropriate [29], and unlike repeated measures ANOVAs, GEE models can incorporate all available data and also model count and binary data. Importantly, GEE estimate population-average parameters and are generally robust to miss-specified correlation structures and overdispersion [30]. In previous similar studies, participants who received other professional help were not included [11] or excluded after randomization [8, 10]. In order to highlight the effects of the web-based intervention in this study, secondary analyses were performed excluding participants in both groups who had received other professional help between baseline and follow-up.

In addition to these analyses, we estimated the effect of intervention adherence on outcomes, using the instrumental variable (IV) approach. This approach first regresses adherence on allocation, and then regresses the outcome on the predicted adherence from the first step [31]. Crucially, unlike per-protocol analyses which provide biased estimates, the IV approach can account for confounding by baseline variables that impact both adherence and outcomes. In this study, we considered number of participant comments (scaled by root mean squared division) and number of completed modules as two separate adherence measures, in separate models. The baseline score of the outcome measure in question was used to control for confounding in each model. IV calculations were performed using the lavaan R package [32], with robust standard errors to account for non-normality, completer data only, and modeled covariance between adherence and outcome measures.

## Results

### Baseline characteristics of study participants

Individuals who met inclusion criteria but did not create an account or complete the baseline assessment (n = 495) were younger [M = 25.8 (SD = 6.9) vs. M = 27.4 (SD = 7.2); t_(796)_ = − 3.029, p = .003], less motivated [M = 66.5 (SD = 30.8) vs. M = 73.8 (SD = 25.0); t_(796)_ = − 3.529, p < .001] and had lower score on CAST [M = 13.1 (SD = 4.9) vs. M = 14.1 (SD = 4.3); t_(796)_ = − 2.837, p = .005] and fewer DSM-5 criteria [M = 7.4 (SD = 2.6) vs. M = 8.1 (SD = 2.1); t_(795)_ − 3.902, p < .001] but did not significantly differ on cannabis use (frequency last 6 months, days without use and quantity in grams during the last 7 days) or demographic questions, compared to participants that were randomized. No statistically significant differences in baseline characteristics, including use of cannabis or alcohol, motivation to change cannabis use, symptoms of depression, and, symptoms of anxiety, between participants in the intervention and control groups where identified, as shown in Table 1.

**Table 1:** Comparison of baseline characteristics among participants in intervention and control groups

	Intervention group (n = 151)	Control group (n = 152)	Test value	p-value
Women (%)	37.7	27.6	χ^2^_(1)_ = 3.524	0.60
Mean age (sd)	27.7 (7.8)	27.1 (6.5)	t_(301)_ = − 0.699	0.485
Civil state				
Single (%)	58.9	53.9	χ^2^_(4)_ = 4.933	0.296
Partner, not married (%)	22.5	28.9		
Married/registered partnership (%)	13.2	14.5		
Divorced/separated (%)	5.3	2.0		
Widow/widower (%)	0	0.7		
Financial situation				
Employment (%)	60.3	63.8	χ^2^_(7)_ = 6.662	0.465
Study allowance (%)	19.2	15.1		
Pension (%)	0.7	0.7		
Sickness/activity compensation (%)	4.6	9.2		
Sickness benefits (%)	3.3	0.7		
Unemployment benefits (%)	1.3	0.7		
Social assistance (%)	4.0	2.6		
Other (%)	6.6	7.2		
Highest completed education				
Unfinished primary school, grade school or equivalent (%)	2.6	6.6	χ^2^_(4)_ = 5.437	0.245
Primary school/grade school (%)	17.9	16.4		
Upper secondary school, vocational school or equivalent (%)	57.6	59.2		
University/College (%)	18.5	17.1		
Other education (%)	3.3	0.7		
Living situation				
Alone (%)	25.8	27.0	χ^2^_(6)_ = 5.399	0.494
With parents (%)	15.2	14.5		
With husband/wife/partner/cohabitant only (%)	12.6	20.4		
With husband/wife/partner/cohabitant and children (%)	9.9	9.9		
With children only	2.6	1.3		
Shifting conditions (%)	28.5	24.3		
Other (%)	5.3	2.6		
Substance use and dependence				
Used cannabis every week in the past 6 months (%)	100.0	100.0		
Cannabis use disorder according to DSM-5 (%)	100.0	100.0		
Used other illicit drugs (%)	85.4	84.9	χ^2^_(1)_ = 0.019	0.891
Hazardous alcohol use (%)	16.6	13.2	χ^2^_(1)_ = 0.692	0.406
Help-seeking				
Sought professional help/treatment for cannabis use in the past 12 months (%)	14.6	13.2	χ^2^_(1)_ = 0.126	0.722
Talked to relatives or friends about reducing or ending cannabis use in the past 12 months (%)	79.5	78.3	χ^2^_(1)_ = 0.063	0.801
Motivation				
Motivation to change cannabis use, mean score on a visual analog scale (VAS) 0–100 (sd)	73.5 (25.5)	74.2 (24.5)	t_(301)_ = 0.232	0.816
VAS score: 0–25 (%)	6.6	6.6	χ^2^_(3)_ = 7.667	0.053
VAS score: 26–50 (%)	13.2	9.2		
VAS score: 51–75 (%)	19.9	33.6		
VAS score: 76–100 (%)	60.3	50.7		
Anxiety and depression				
Moderate or severe anxiety GAD-7 ≥ 10 (%)	41.7	39.5	χ^2^_(1)_ = 0.159	0.690
Moderate or depression MADRS ≥ 20 (%)	62.3	58.6	χ^2^_(1)_ = 0.433	0.510

### Follow-up completers

There was a significant difference in the number of participants in each group that completed the 3-mount follow-up (χ^2^_(1)_ = 10.288, p = .001). Several statistically significant differences in baseline characteristics between participants who completed the 3-month follow-up (n = 128) and participants who did not respond to the follow-up invitation (n = 175) were found. In comparison to the non-completers, study participants who completed the follow-up were older [M = 28.6 (SD = 7.5) vs. M = 26.6 (SD = 6.9); t_(301)_ = − 2.414; p = .016], had more frequently completed university or college (23.4% vs. 13.7%; χ^2^_(1)_ = 4.772; p = .029) and lived less frequently with their parents (7.8% vs. 20.0%; χ^2^_(1)_ = 8.683; p = .003). In addition, on average, they reported fewer cannabis syndrome criteria [M = 7.6 (SD = 2.2) vs. 8 M = .4 (SD = 2.0); t_(300)_ = 3.599; p < .001], used less cannabis per week [M = 5.0 (SD = 4.1) vs. M = 6.1 (SD = 4.4); t_(294)_ = − 2.030; p = .043], had lower scores for anxiety [M = 8.2 (SD = 5.4) vs. M = 9.5 (SD = 5.4); t_(301)_ = 2.106; p = .036] and had lower scores on CAST [M = 13.1 (SD = 4.4) vs. M = 14.8 (SD = 4.1); t_(301)_ = 3.349; p < .001].

### Reasons for using internet intervention and utilization of the intervention

The most endorsed reason for using internet-based intervention was to be able to remain anonymous. See Table 2 for details. Utilization of the web-based treatment program is described in Table 3. In the intervention group n = 53 (35%) never visited the treatment again after the 1st day. Of the 13 modules in the program, participants in the intervention group completed on average M = 3.9 (SD = 2.7) modules and visited the treatment on average M = 65.9 (SD = 112.7) days. They also conducted an average of M = 13.8 (SD = 17.7) calendar registrations and wrote an average of M = 6.5 (SD = 8.0) personal comments. Only 12 of the participants in the control group started the program when they were offered, after they had completed the follow-up.

**Table 2:** Reasons for choosing internet-based treatment

	N	Mean^a^	SD
Can remain anonymous	256	8.1	2.9
Can decide my own goal within the treatment	255	7.8	2.7
Do not have to tell other people that you are seeking treatment	256	7.3	3.2
Do not have to travel to participate in treatment	253	7.1	3.2
Can have access to treatment at any time	254	7.0	2.9
Cannabis use is not documented in a medical record	256	6.6	3.8
Do not have to go to a clinic	254	6.0	3.8

^a^On a scale from 0—not at all important to 10—very important

**Table 3:** Utilization of Cannabishjälpen program in the intervention group (n = 151)

Step (days since last use)	Visited step page^a^	Module^b^	Visited module page	Completed module
Step 0 (0–1 day)	82 (53.9%)	Finding motivation to change cannabis use	102 (67.5%)	90 (59.6%)
		Setting a goal of a time period when not to use cannabis	85 (56.3%)	78 (51.7%)
		Mean (SD) goal in number of cannabis abstinent days^c^		62.6 (68.1)
Step 1 (2–8 days)	50 (32.9%)	Learning practical tips on changing cannabis use through self-control	68 (45.0%)	57 (37.7%)
		Picturing yourself free from cannabis	49 (32.5%)	28 (18.5%)
		How cannabis affects your thinking	26 (17.2%)	20 (13.2%)
Step 2 (9–21 days)	31 (20.4%)	Alternative ways to manage cravings	33 (21.9%)	22 (14.6%)
		Finding ways to sleep better	32 (21.2%)	22 (14.6%)
		Learning to deal with difficult emotions	25 (16.6%)	17 (11.3%)
		Practicing the handling of social pressure	18 (11.9%)	15 (9.9%)
Step 3 (22–42 days)	23 (15.1%)	Learning to get help from others	11 (7.3%)	9 (6.0%)
		Identifying risk situations that trigger the urge to use cannabis	15 (9.9%)	1 (0.7%)
		Alternative plans for handling problems	15 (9.9%)	12 (7.9%)
		Relapse prevention (including tips on maintaining motivation to sustain abstinence from cannabis use over the long term)	9 (6.0%)	6 (4.0%)
Introduction (all)	137 (90.7%)	Used the cannabis calendar		96 (63.6%)
		Sent a message to therapist		76 (50.3%)
		Mean (SD number of visits to the program		62.4 (110.6)
		Mean (SD) days between first and last visit		25.0 (33.6)

^a^The step pages (0–3) included psychoeducation on what the user could expect during the specific period and some brief suggestions

^b^Modules where recommended automatically based on the number of days since the user smoked. All modules where available from start

^c^Recommended goal for cannabis abstinence was 6 weeks (42 days)

### Time by group effects

In the ITT analyses, GEE models revealed no time by group effects on any outcome, with a trend (p = .07) observed on CAST scores. See Table 4 for full results. In the secondary analysis, excluding participants who sought other treatment, significant time by group effects were found for gram cannabis past week, number of dependency criteria and CAST-score. Main effects of time were observed on several outcomes, revealing that lack of interaction effects could be attributed to change also in the control group.

**Table 4:** Observed means and outcome model parameters

Intention-to-treat analyses (n = 303)													
Outcome	Pre M (SD) or n (%)				Post M (SD) or n (%)				Outcome modelling				
	Intervention		Control		Intervention		Control		Type	Intercept	Time	Group	Time × group
Number of days without cannabis use (past week)	1.60	(2.03)	1.38	(1.94)	4.16	(2.74)	3.27	(2.70)	Poisson	B = 0.32 (SE = 0.11, p < .001)	B = 0.85 (SE = 0.14, p < .001)	B = 0.14 (SE = 0.15, p = .35)	B = 0.09 (SE = 0.19, p = .63)
Gram cannabis (past week)	5.98	(5.64)	6.87	(7.99)	2.30	(2.92)	4.06	(6.69)	Poisson	B = 1.93 (SE = 0.09, p < .001)	B = − 0.45 (SE = 0.11, p < .001)	B = − 0.14 (SE = 0.12, p = .25)	B = − 0.27 (SE = 0.18, p = .12)
Number of DSM-5 cannabis use disorder criteria	7.93	(2.22)	8.18	(2.05)	5.02	(2.61)	5.74	(2.69)	Gaussian	B = 8.18 (SE = 0.17, p < .001)	B = − 2.17 (SE = 0.28, p < .001)	B = − 0.25 (SE = 0.25, p = .31)	B = − 0.61 (SE = 0.48, p = .2)
CAST score	14.05	(4.33)	14.07	(4.31)	8.26	(5.10)	9.73	(5.37)	Gaussian	B = 14.07 (SE = 0.35, p < .001)	B = − 3.8 (SE = 0.53, p < .001)	B = − 0.02 (SE = 0.49, p = .97)	B = − 1.63 (SE = 0.9, p = .07)
Help-seeking (n, %)	17	(11.26)	19	(12.5)	12	(24)	17	(21.79)	Binomial	B = − 1.95 (SE = 0.25, p < .001)	B = 0.56 (SE = 0.28, p = .05)	B = − 0.12 (SE = 0.36, p = .74)	B = 0.25 (SE = 0.49, p = .61)
Number of standard glasses of alcohol (past week)	6.29	(8.03)	5.62	(8.11)	4.28	(5.11)	5.44	(9.45)	Poisson	B = 1.73 (SE = 0.12, p < .001)	B = − 0.09 (SE = 0.2, p = .65)	B = 0.11 (SE = 0.16, p = .47)	B = − 0.26 (SE = 0.25, p = .3)
SCS score	48.87	(13.26)	49.05	(14.52)	54.10	(14.70)	54.77	(15.84)	Gaussian	B = 49.05 (SE = 1.17, p < .001)	B = 4.83 (SE = 1.34, p < .001)	B = − 0.17 (SE = 1.59, p = .91)	B = 0.17 (SE = 2.22, p = .94)
MADRS score	21.62	(8.91)	20.92	(9.79)	16.78	(10.99)	16.22	(11.27)	Gaussian	B = 20.92 (SE = 0.79, p < .001)	B = − 4.11 (SE = 0.94, p < .001)	B = 0.69 (SE = 1.07, p = .52)	B = − 0.67 (SE = 1.68, p = .69)
GAD-7 score	9.28	(5.50)	8.67	(5.34)	6.60	(5.57)	6.36	(5.52)	Gaussian	B = 8.67 (SE = 0.43, p < .001)	B = − 1.97 (SE = 0.52, p < .001)	B = 0.61 (SE = 0.62, p = .33)	B = − 0.19 (SE = 0.85, p = .82)

Excluding participants who sought other treatment (n = 267)													
	Pre M (SD) or n (%)				Post M (SD) or n (%)				Outcome modelling				
	Intervention		Control		Intervention		Control		Type	Intercept	Time	Group	Time × group
Number of days without cannabis use (past week)	1.52	(1.99)	1.29	(1.83)	4.26	(2.68)	3.16	(2.48)	Poisson	B = 0.32 (SE = 0.12, p = .01)	B = 0.83 (SE = 0.15, p < .001)	B = 0.05 (SE = 0.17, p = .76)	B = 0.27 (SE = 0.21, p = .2)
Gram cannabis (past week)	5.82	(5.53)	6.99	(8.18)	2.03	(2.60)	4.05	(6.96)	Poisson	B = 1.95 (SE = 0.1, p < .001)	B = − 0.42 (SE = 0.13, p < .001)	B = − 0.12 (SE = 0.13, p = .36)	B = − 0.42 (SE = 0.19, p = .02)
Number of DSM-5 cannabis use disorder criteria	7.79	(2.26)	8.04	(2.03)	4.42	(2.46)	5.87	(2.50)	Gaussian	B = 8.11 (SE = 0.18, p < .001)	B = − 2.06 (SE = 0.3, p < .001)	B = − 0.26 (SE = 0.26, p = .32)	B = − 1.36 (SE = 0.52, p = .01)
CAST score	13.62	(4.26)	13.90	(4.24)	7.79	(5.07)	9.80	(5.10)	Gaussian	B = 14.05 (SE = 0.38, p < .001)	B = − 3.45 (SE = 0.56, p < .001)	B = − 0.15 (SE = 0.53, p = .78)	B = − 1.9 (SE = 0.99, p = .06)
Number of standard glasses of alcohol (past week)	6.48	(7.97)	5.62	(8.08)	4.92	(5.42)	4.38	(5.48)	Poisson	B = 1.65 (SE = 0.13, p < .001)	B = − 0.22 (SE = 0.16, p = .17)	B = 0.18 (SE = 0.17, p = .27)	B = 0.09 (SE = 0.22, p = .69)
SCS score	49.18	(13.67)	49.74	(14.57)	56.97	(13.14)	55.93	(15.97)	Gaussian	B = 49.78 (SE = 1.27, p < .001)	B = 5.19 (SE = 1.64, p < .001)	B = − 0.54 (SE = 1.71, p = .75)	B = 2.45 (SE = 2.67, p = .36)
MADRS score	21.17	(9.03)	20.48	(9.58)	14.29	(9.98)	16.20	(11.91)	Gaussian	B = 20.36 (SE = 0.86, p < .001)	B = − 3.22 (SE = 1.1, p < .001)	B = 0.93 (SE = 1.14, p = .41)	B = − 3.3 (SE = 1.93, p = .09)
GAD-7 score	8.84	(5.43)	8.17	(5.08)	5.61	(5.18)	5.80	(5.12)	Gaussian	B = 8.09 (SE = 0.44, p < .001)	B = − 2.11 (SE = 0.62, p < .001)	B = 0.89 (SE = 0.63, p = .16)	B = − 0.76 (SE = 0.96, p = .43)

### Estimated effect of intervention adherence

**Fig. 2:**
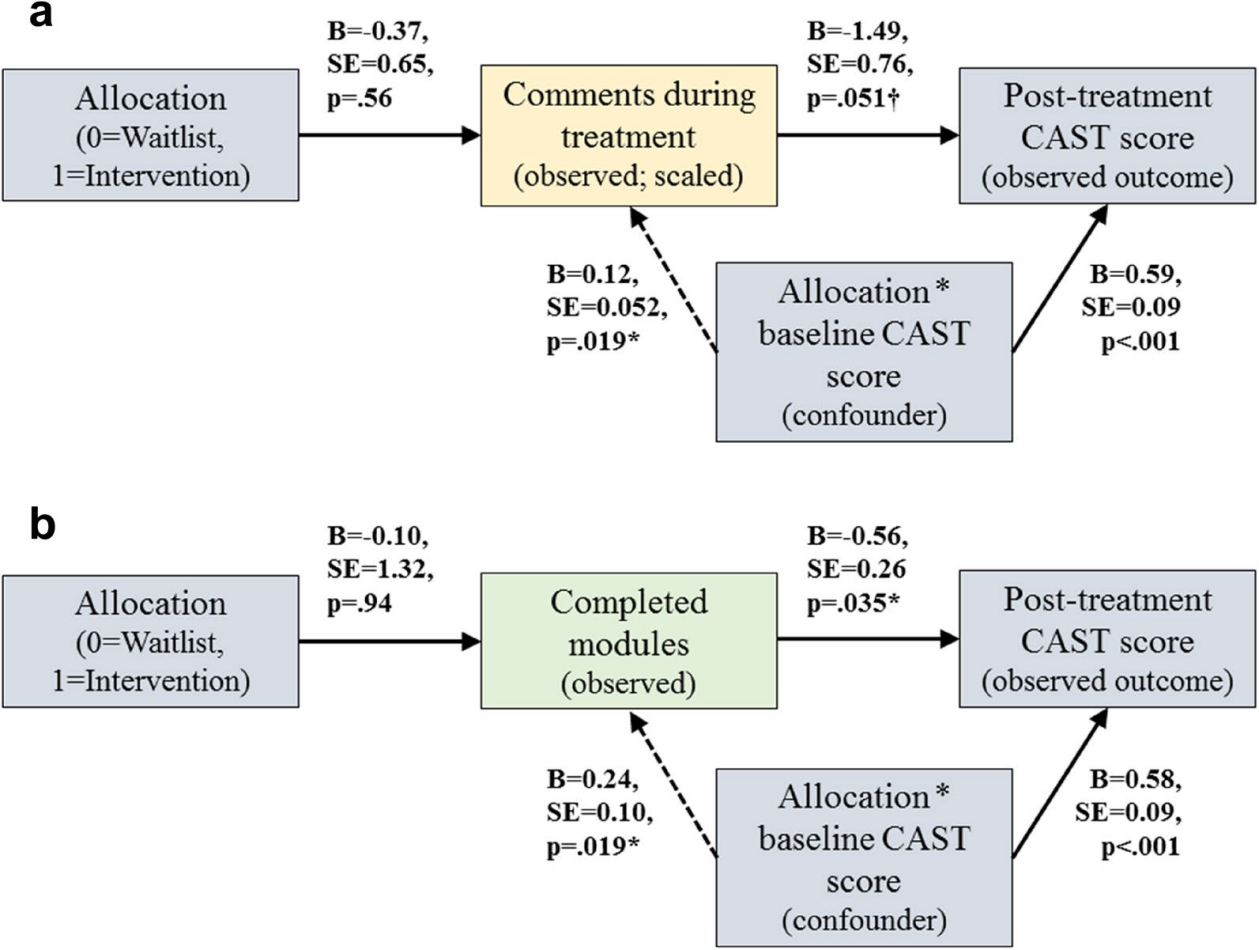
Estimated effect of intervention adherence using instrumental variable approach. **a** Model with comments during treatment as adherence measure. **b** Model with completed modules as adherence measure

Effects of intervention adherence was examined only on difference in CAST scores, since this outcome was the only one to show trend-level time by group effects. Results showed a near-significant (p = .051) negative association between number of posted comments and post-intervention CAST scores, and a significant (p = .035) negative association between number of completed modules and post-intervention CAST-scores, adjusting in both models for significant confounding effects of baseline CAST scores on both adherence (both p = .019) and post-treatment scores. See Fig. 2 for details.

### Help-seeking

Among participants who completed follow-up (n = 128) the degree of help-seeking was not significantly different between intervention and control groups (Professional: n = 17 (21.8%) vs. n = 12 (24.0%), χ^2^_(1)_ = 0.096, p = .757) Participating in the study did not increase help-seeking in any of the groups between baseline and 3-month follow-up.

## Discussion

The main finding from this study is that access to a web-based treatment program with therapist guidance did not lead to significant additional decreases in cannabis use, associated symptoms or increase in help-seeking compared to a waiting list, which also showed improvement over time. The level of help-seeking did not increase significantly more in the intervention group or over time. When participants who received other professional help between baseline and follow-up were removed from the analysis, the intervention group showed significantly greater reductions over time in gram cannabis used past week, number of cannabis use disorder criteria and CAST scores.

The reductions in cannabis use in the intervention group was similar to the changes observed in previous studies [8, 10, 11], but the reduction in cannabis-use in our control group was greater than those in the control group in two of those studies (− 35% vs. − 13 and − 12%) [8, 11]. The changes in our control group could be an effect of the recruiting strategy, which targeted active help-seekers. Motivation to change substance use is an unstable characteristic which often varies considerably over time [33]. All participants in this study were recruited based on their initial level of cannabis-use and they had high readiness to change (74 out of 100). Some of the decrease could be explained by regression towards the mean [34]. All participants also answered a large amount of assessment questions about their cannabis use and that in it-self can lead to reductions in substance use [35, 36]. The lack of statistical significance in difference between groups in the ITT analysis may also be due in part to inadequate power, resulting from not only larger-than-expected change in the waiting-list group, but also attrition.

Our findings also suggest that adherence to the program is associated with lower cannabis use disorder symptoms at follow-up, in analyses that correct for baseline confounding on both adherence and outcomes. Interestingly, adherence was positively associated with pre-treatment scores, revealing that participants with greater baseline severity engaged more in the intervention and also benefitted more from it. These findings suggest that the intervention may benefit from including more adherence-promoting actions, targeting specific groups of users. Whether this leads to greater adherence and indirectly better outcomes will need to be evaluated in a future study.

### Limitations and strengths

One limitation in this study is the large attrition rate at follow-up. Although, low follow-up rate is a common occurrence in studies investigating the effects of internet-based interventions for problematic substance use [37] and similar to previous studies on cannabis [8, 10, 11], it constitutes a methodological problem in so far as power decreases and results are harder to interpret or generalize to the broader population of help-seekers. Future studies should include weekly measures to capture when change occurs during treatment and allow statistical methods that can estimate missing data [29]. Yet another limitation of the current study is the relatively short follow-up period. Determining the follow-up period is a balancing act between methodological design and the ethical issue of having participants randomized to a wait-list control group for extended periods, while having identified problematic cannabis use but not being offered immediate help for this. Participants in this study were blinded to group allocation as well as waiting times which hopefully reduced the risk of being negatively affected by being allocated to the control group [38, 39].

This study contributes to the research field and adds value in terms of increased understanding of whether and how internet can be used to reach individuals with comprehensive and problematic use of cannabis. Further, this study increases our understanding of characteristics of individuals primarily interested in treatment via an internet-based platform. This is important in order to design more effective internet-based interventions in the future. In addition, this knowledge is important in order to improve study designs to obtain more reliable results regarding effectiveness of internet-based interventions. Results from this study adds some support to previous research indicating internet-based treatment program with therapist support or guidance can be effective in reducing severe cannabis use and associated negative consequences.

### Future research

More RCTs are needed to build an evidence base for the effectiveness of internet-based interventions for reducing cannabis use and, in the long run, to investigate the effects of a range of content and features in such interventions. The results of the current study are limited to the help-seeking population of cannabis-users. Studies targeting all cannabis-users in a population might increase generalizability but could lead to lower retention in treatment, since many cannabis users are not interested in changing their habits. More studies are needed to increase our understanding of how such interventions are used by consumers and why so many do not utilize the content in internet-based interventions on more occasions. Further, we need a greater understanding of the underlying reasons for the large attrition in studies investigating effects of internet-based interventions for reducing substance-use. Studies in better controlled settings might address these problems but could also affect the population studied. Increased demands on users to identify themselves or have contact with a health-professional or research-assistant might make substance users who wish to stay anonymous, more reluctant to enter a study. Finally, increased knowledge about user preferences regarding content, function and design of internet-based interventions, as well as to determine what aspects of such interventions consumers perceive as unimportant, may provide guidance in the development of more engaging or more effective intervention designs in the future.

## Conclusions

The current study found no evidence in favor of a web-based treatment program with therapist guidance over waiting-list in decreasing cannabis use or associated symptoms. The web-based treatment program was however successful in reaching individuals with extensive and regular cannabis use who had low rates of help-seeking. In secondary analyses including only those who had not received professional help, the treatment program led to greater reductions in use and symptoms. The latter findings should however be interpreted with caution.

## Data Availability

The datasets analyzed during the current study and the web-based intervention are available from the corresponding author on reasonable request.
